# Clinical and Immunological Recovery Trajectories in Severe COVID-19 Survivors: A 12-Month Prospective Follow-Up Study

**DOI:** 10.3390/v17121610

**Published:** 2025-12-12

**Authors:** Edita Strumiliene, Laura Malinauskiene, Jurgita Urboniene, Laimutė Jurgauskienė, Birutė Zablockienė, Ligita Jancoriene

**Affiliations:** 1 Clinic of Infectious Diseases and Dermatovenerology, Institute of Clinical Medicine, Faculty of Medicine, Vilnius University, LT-01513 Vilnius, Lithuania; 2 Clinic of Chest Diseases, Immunology and Allergology, Institute of Clinical Medicine, Faculty of Medicine, Vilnius University, LT-01513 Vilnius, Lithuania; 3 Centre of Infectious Diseases, Vilnius University Hospital Santaros Klinikos, LT-08406 Vilnius, Lithuania; 4 Clinic of Cardiac and Vascular Diseases, Institute of Clinical Medicine, Faculty of Medicine, Vilnius University, LT-01513 Vilnius, Lithuania

**Keywords:** long COVID, immune recovery, complement, NK cells, post-acute sequelae

## Abstract

**Background:** The link between clinical recovery and immune restoration after severe COVID-19 remains poorly defined. Although most survivors experience symptomatic improvement, persistent symptoms have been hypothesized to reflect ongoing immune dysregulation. **Methods:** This prospective cohort study followed 93 unvaccinated adults with RT-PCR-confirmed moderate-to-critical COVID-19 at 3, 6, and 12 months post-discharge. Clinical assessments used structured interviews to evaluate the persistent symptoms. Peripheral blood analyses were used to measure lymphocyte subsets, immunoglobulins, and complement components. **Results:** Clinical recovery was substantial; fatigue prevalence declined from 70.9% to 24.7% and dyspnea prevalence from 81.7% to 25.8% by 12 months (*p* < 0.001 for both). However, immune recovery exhibited divergent patterns. Activated T cells (CD3^+^HLA-DR^+^) decreased significantly (from 20% to 13%; *p* < 0.001), complement C3c levels paradoxically increased from 1.23 to 1.35 g/L (*p* < 0.001), and serum IgA increased by 32% (*p* = 0.003). NK cells remained stable overall but were persistently reduced in a subset (~25%) of patients, particularly among those with fatigue and dyspnea. Critical illness was associated with slower T-cell resolution, prolonged IgM elevation, and increased complement activity. **Conclusions:** One year after hospitalization, most patients achieved substantial clinical improvement, but immune reconstitution lagged behind. These findings highlight the dissociation between clinical and immunological recovery and suggest that persistent immune dysregulation may be associated with long COVID manifestations. Incorporating immune monitoring into post-COVID care may help identify patients at risk of prolonged sequelae and guide targeted therapeutic strategies.

## 1. Introduction

Since its emergence in late 2019, coronavirus disease 2019 (COVID-19) has imposed a substantial and ongoing global health burden. Although advances in vaccination, antiviral therapy, and supportive care have improved acute outcomes, the long-term consequences of SARS-CoV-2 infection remain a major clinical and public health challenge. A growing body of evidence highlights the post-acute sequelae of SARS-CoV-2 infection (PASC), or long COVID, a heterogeneous condition characterized by symptoms that persist or recur beyond three months after the acute illness [1,2,3]. These manifestations—most commonly fatigue, dyspnea, reduced exercise tolerance, neurocognitive difficulties, and musculoskeletal complaints—may significantly impair daily functioning and quality of life [4,5,6,7,8,9,10,11].

Severe COVID-19 is associated with marked immune dysregulation, including lymphopenia, excessive T-cell activation, impaired innate responses, and disturbances in humoral and complement pathways [4,6]. Although partial immune restoration occurs during convalescence, accumulating data indicate that certain immune alterations may persist for months after viral clearance. Complement activation, particularly at the C3 level, has been linked to endothelial injury, microvascular dysfunction, and worse outcomes during acute disease [12,13], yet its long-term trajectory in survivors remains poorly understood. Persistent mucosal immune engagement and sustained IgA responses have also been described, suggesting that some aspects of humoral immunity may remain activated long after recovery [14,15,16,17].

Despite increasing recognition of long-term immunological perturbations, prospective studies that integrate detailed immune profiling with longitudinal clinical assessment are limited. Most available data focus on early recovery (3–6 months after infection), leaving the 12-month immune reconstitution trajectory—and its relationship with persistent symptoms—insufficiently defined.

To address these gaps, we conducted a 12-month prospective follow-up study of previously unvaccinated adults hospitalized with moderate-to-critical COVID-19. Our objectives were to:

(i) characterize longitudinal changes in lymphocyte subsets, activation markers, NK cells, immunoglobulins, and complement components;
(ii) examine associations between immune recovery and persistent clinical symptoms; and
(iii) explore whether immune trajectories differ according to the initial severity of illness.

## 2. Materials and Methods

### 2.1. Study Design and Participants

This prospective cohort study was conducted at Vilnius University Hospital Santaros Klinikos, a tertiary COVID-19 referral center in Lithuania. Adults with RT-PCR–confirmed SARS-CoV-2 infection who were hospitalized between October 2021 and October 2022 were invited to participate in the study upon discharge. COVID-19 severity was classified as moderate, severe, or critical according to the World Health Organization (WHO) guidelines [18].

Exclusion criteria were active malignancy, known primary or secondary immunodeficiency, inability to provide informed consent, or refusal to participate.

All participants were unvaccinated at the time of acute illness and during follow-up, offering a unique perspective on immune recovery in the absence of vaccine-induced modulation.

### 2.2. Follow-Up Schedule and Clinical Data Collection

Participants attended study visits at 3, 6, and 12 months after hospital discharge. Each visit included:

- A structured interview to assess persistent symptoms (fatigue, dyspnea, exercise intolerance, neurocognitive complaints, musculoskeletal symptoms, and others); participants were asked whether each symptom was new compared with their pre-COVID condition. Only new or worsened symptoms were recorded as post-COVID manifestations according to the WHO clinical case definition (symptoms persisting ≥ 3 months and not explained by alternative diagnoses),
- All participants were asked about immunomodulatory medication use during the 12-month follow-up,
- A targeted clinical evaluation,
- Peripheral blood sampling for immunological analysis.

Baseline demographic characteristics, comorbidities, and acute COVID-19 illness details (including ICU admission, need for mechanical ventilation, and in-hospital complications) were extracted from electronic health records.

Body mass index (BMI) was calculated as weight (kg)/height^2^ (m^2^) and categorized per WHO criteria [19].

### 2.3. Laboratory Assessments

Peripheral blood samples were collected in EDTA tubes at 3, 6, and 12 months post-discharge and processed within 4 h using standardized protocols.

Reference Ranges

Hospital laboratory reference ranges were applied for all parameters:

Cellular immunity: Total lymphocytes 1100–2400 cells/mm^3^ (25–39%); CD3^+^ T cells 1100–1700/mm^3^ (67–76%); CD3^+^CD4^+^ helper T cells 700–1100/mm^3^ (38–46%); CD3^+^CD8^+^ cytotoxic T cells 500–900/mm^3^ (31–48%); CD4/CD8 ratio 1.0–2; NK (CD16^+^CD56^+^) cells 200–400 cells/mm^3^ (10–19%); CD19^+^ B cells 200–400 cells/mm^3^ (5–18%); CD3^+^HLA-DR^+^ activated T cells < 25%.

Humoral immunity: IgA 0.7–4.0 g/L; IgG 7.0–16.0 g/L; IgM 0.4–2.3 g/L; IgE 0–100 kU/L; complement C3c 0.9–1.8 g/L; complement C4 0.1–0.4 g/L.

Flow Cytometry Analysis

Analysis of white blood cells, total lymphocyte counts, and subpopulations CD3^+^, CD3^+^CD4^+^, CD3^+^CD8^+^, CD4/CD8 ratio, NK cells (CD16^+^CD56^+^), B lymphocytes (CD19^+^) and activated T cells (CD3^+^HLA-DR^+^) was performed using direct flow cytometry.

Samples were acquired on a FACSCalibur flow cytometer (BD Biosciences, San Jose, USA.) with minimum 10,000 events per sample and analyzed using CellQuest Pro software (Version 5.2.1). Analysis regions were gated using CD45/CD14 antibody combination to identify leukocytes, with isotype controls (γ1/γ2a) for negative marker settings. Forward and side scatter gating excluded debris and doublets.

Absolute lymphocyte subset numbers were calculated based on total WBC from hematology analyzer and lymphocyte percentage, adjusted to subset percentages determined by flow cytometry. Quality control was maintained through daily calibration with calibration beads and participation in external quality assurance programs.

Immunoglobulin and Complement Analysis

Serum levels of immunoglobulins (IgA, IgG, IgM, IgE) and complement components (C3c, C4) were measured using nephelometry on a BN analyzer (Siemens Healthcare Diagnostics, Erlangen, Germany) according to manufacturer instructions. All measurements were performed in duplicate, and quality control was ensured through daily calibration and participation in external quality assurance programs.

Reference Ranges and Quality Control

All assays were performed according to manufacturer instructions by experienced laboratory personnel blinded to clinical data and patient outcomes. Reference ranges established by the hospital’s central laboratory were applied for all parameters. Internal quality control samples were analyzed with each batch to ensure consistency and accuracy.

### 2.4. Missing Data Analysis

Not all laboratory assessments were available at each visit due to missed appointments, insufficient sample volumes, or technical failures. Missingness was evaluated using Little’s MCAR test, and analyses were restricted to participants with complete data for the variables of interest. No imputation was applied to preserve data integrity. Internal and external quality assurance procedures were in place for all assays.

For symptom-laboratory correlations, analyses were restricted to participants with complete data for specific parameters being compared. Denominators for each analysis are clearly specified in result tables. No imputation was performed given the observational study nature and requirement for precise laboratory measurements.

### 2.5. Statistical Analysis

Continuous variables were summarized as median (interquartile range, IQR), and categorical variables as frequencies (%). The Shapiro–Wilk test was used to assess normality. Group comparisons included McNemar’s test for within-subject changes in symptom prevalence, Wilcoxon signed-rank or Mann–Whitney U tests for continuous variables, and χ^2^ test or Fisher’s exact test for categorical variables. A two-sided *p*-value < 0.05 was considered statistically significant.

Unsupervised clustering (Gower distance; agglomerative Ward on PCoA) was performed to map symptoms to immune patterns. Symptoms were binary in three domains—respiratory (dyspnea, reduced exercise tolerance), neurocognitive (cognitive dysfunction/memory impairment), musculoskeletal (myalgia/arthralgia). Immune features (CD16^+^CD56^+^/%, CD3^+^%, CD3^+^CD4^+^%, CD3^+^CD8^+^%, CD4/CD8 ratio, CD19^+^, CD3^+^HLA-DR^+^%, C3c, C4, IgA, IgM) were z-scored and summarized at 3 and 6 months; 12-month values informed sensitivity analyses. Cluster number was chosen by dendrogram/elbow/silhouette; stability by 1000 bootstraps (Jaccard). Between-cluster differences used Kruskal–Wallis (Dunn–Bonferroni) and Fisher’s exact with BH-FDR control (*p* < 0.10). Sensitivity: (i) 6-month features only; also, sensitivity analyses included (ii) covariate-residualized immune features, in which each marker was adjusted for age, sex, BMI, and initial COVID-19 severity before clustering.

Heatmaps were used to display effect sizes, defined as the difference between group medians divided by the cohort interquartile range (IQR) at the same timepoint. A diverging color scale centered at 0 facilitated comparisons across markers and timepoints.

Analyses were performed using IBM SPSS v20.0, and visualizations were created in GraphPad Prism v8.0.

### 2.6. Ethical Considerations

The study was approved by the Vilnius Regional Biomedical Ethics Committee (Protocol No. 2020/6-1233-718, 22 June 2020) and conducted in accordance with the Declaration of Helsinki [20]. Written informed consent was obtained from all participants before study inclusion, including consent for publication of anonymized data.

Datasets generated during this study are available from the corresponding author upon reasonable request, subject to appropriate ethical approval and data sharing agreements.

## 3. Results

### 3.1. Study Population and Follow-Up Completion

Of 100 enrolled patients, 93 completed all follow-up visits and were included in the final analysis. The cohort comprised 50 women (53.8%) and 43 men (46.2%) with a median age of 59 years (IQR 51–64.5). Comorbidities were present in 59 patients (63.4%). Most participants were overweight or obese (91.4%), and all were unvaccinated at the time of infection and throughout follow-up. Regarding disease severity, 17 patients (18.3%) had moderate, 32 (34.4%) severe, and 44 (47.3%) critical COVID-19. ICU admission was required for 43 patients (46.2%), with a median stay of 12 days (IQR 8–16); 7 patients (7.5%) underwent invasive mechanical ventilation. In-hospital complications occurred in 34 patients (36.6%). Laboratory data completeness varied across visits due to missed appointments and technical factors, with sample sizes ranging from 82 to 93 patients per parameter and timepoint (detailed each table separately).

No patient received long-term corticosteroids, immunomodulatory agents, or antivirals after discharge.

### 3.2. Symptom Recovery over Time

At 3 months, the majority reported fatigue (70.9%), dyspnea (81.7%), and reduced exercise tolerance (71.0%). These core symptoms declined substantially by 12 months: fatigue to 24.7%, dyspnea −25.8%, and reduced exercise tolerance −24.7% (all *p* < 0.001). This represents a two-thirds reduction in prevalence (Table 1).

Other symptoms such as musculoskeletal pain, cardiac complaints, insomnia/anxiety, and cognitive dysfunction and memory impairment were less frequent. Of these, only cardiac symptoms showed a significant early decline (11.8% at 3 months vs. 2.2% at 6 months, *p* = 0.004).

**Figure 1:**
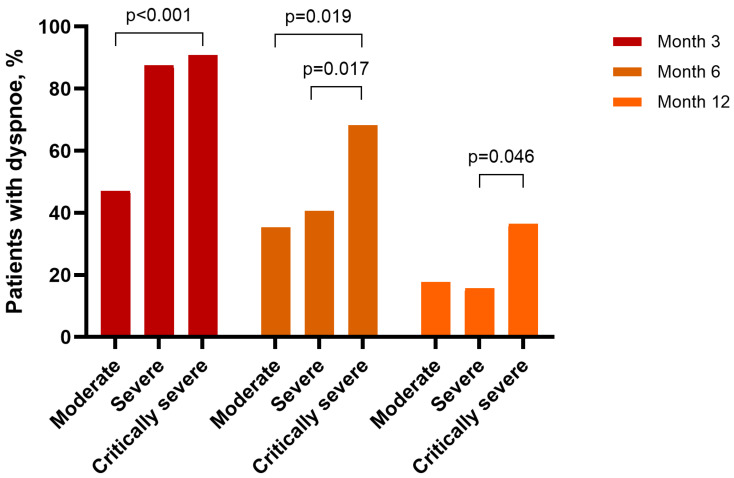
Prevalence of dyspnea at 3, 6, and 12 months after discharge, stratified by initial COVID-19 severity. The figure displays changes in dyspnea prevalence over time in patients with moderate, severe, and critical illness.

Symptom persistence was influenced by initial severity: at 12 months, dyspnea persisted in 36.4% of critically ill patients, compared to 17.6% of moderate cases (*p* = 0.025; Figure 1). Reduced exercise tolerance followed a similar pattern (34.1% vs. 5.9%).

### 3.3. Immune System Recovery

#### 3.3.1. Cellular Immunity

Total lymphocyte counts remained within the normal range throughout follow-up. However, immune activation markers demonstrated dynamic changes:

- Activated T cells (CD3^+^HLA-DR^+^) declined from 20% at 3 months to 13% at 12 months (*p* < 0.001).
- CD3^+^CD8^+^ T cells decreased significantly by 12 months (572 vs. 632 cells/µL at 3 months, *p* = 0.020).
- The CD4/CD8 ratio showed a gradual upward trend, suggesting immune homeostasis restoration.

By contrast, NK cells and B cells remained stable at the group level. However, a subset (~20%) of patients demonstrated persistently low NK counts across all visits (Table 2).

#### 3.3.2. Complement Activation and Immunoglobulin Changes

C3c Demonstrates Sustained Elevation

The most striking finding was the progressive increase in complement C3c levels throughout the 12-month follow-up period, occurring paradoxically as clinical symptoms improved (Table 3). Complement C3c levels rose progressively from 1.23 g/L at 3 months to 1.35 g/L at 12 months (+10%, *p* < 0.001), despite clinical improvement. C4 showed only minor transient fluctuations.

Progressive IgA Elevation Suggests Mucosal Immune Activation

IgA levels demonstrated significant and sustained increase throughout follow-up, rising from 2.06 g/L (IQR 1.53–2.67) at month 3 to 2.42 g/L (IQR 1.81–3.74) at month 6 (*p* < 0.001), and remaining elevated at 2.72 g/L (IQR 1.82–3.35) at month 12 (*p* = 0.003 vs. month 3). In contrast, IgM declined modestly, while IgG and IgE remained stable (Table 3).

### 3.4. Clinical-Immunological Correlations

Immune Markers and Persistent Symptoms

Several statistically significant associations were identified between immune parameters and persistent post-COVID symptoms, revealing potential mechanistic links between immune dysfunction and symptom persistence.

**Figure 2:**
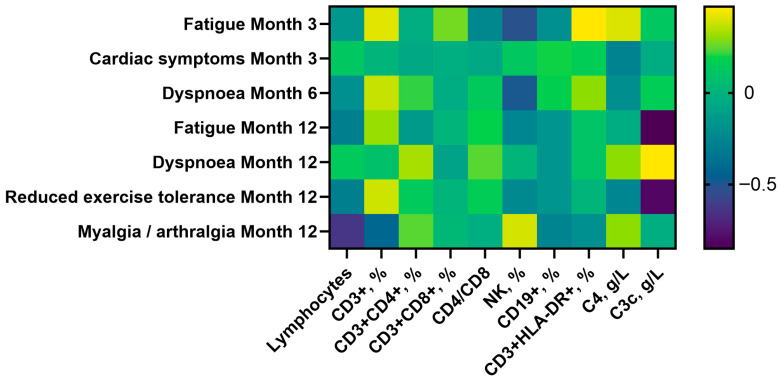
Heatmap of immune marker effect sizes across symptom groups and timepoints. Effect sizes indicate median differences standardized by cohort IQR, illustrating associations between immune features and persistent symptoms.

Exploratory analyses identified several associations (Figure 2):

- Fatigue (3 months): linked to lower NK counts (293 vs. 478 cells/µL, *p* = 0.005) and higher CD3^+^ T-cell percentages (72% vs. 68%, *p* = 0.029).
- Dyspnea (6 months): associated with reduced NK counts (240 vs. 426 cells/µL, *p* = 0.006) and higher CD3^+^CD4^+^ T-cell percentages (40% vs. 38%, *p* = 0.045).
- Myalgia/arthralgia (12 months): associated with lower lymphocyte, CD3^+^, CD3^+^CD4^+^, and CD19^+^ cell counts.
- Cardiac symptoms (3 months): associated with higher C3c (1.37 vs. 1.20 g/L, *p* = 0.026).

Interestingly, at 12 months, patients with fatigue and reduced exercise tolerance had lower C3c compared to asymptomatic peers, suggesting a complex role of complement in long COVID.

In summary, although the limited sample size precluded formal correlation analysis, exploratory comparisons indicated that patients reporting persistent fatigue and dyspnea at 12 months exhibited higher levels of activated T cells (CD3^+^HLA-DR^+^), slightly elevated C3c concentrations, and lower CD3^+^CD8^+^ T cell counts compared with fully recovered individuals. These preliminary trends suggest that sustained immune activation may underlie lingering post-COVID symptoms, warranting further investigation in larger cohorts.

Symptom clusters and immune endotypes

Unsupervised clustering of participants with complete symptom and immune data consistently resolved three clinically interpretable clusters (endotypes) across bootstrap resamples. The clusters aligned with the pre-specified symptom domains and distinct immune signatures observed in our primary analyses:

Endotype A—Respiratory (“Complement/NK-suppressed”)

Characterized by dyspnea and reduced exercise tolerance, this cluster showed lower NK-cell counts/percentages and higher CD3/CD4 at 6 months, consistent with impaired innate cytotoxicity alongside persistent adaptive activation. Patients with early cardiopulmonary complaints (month 3) also exhibited higher C3c, linking this endotype to complement activity; conversely, by 12 months fatigue/reduced exercise tolerance coincided with lower C3c, suggesting phase-specific or heterogeneous complement dynamics. IgM was higher at 6 months among those with dyspnea, indicating ongoing antigenic stimulation.

Endotype B—Neurocognitive (“Neuro–T-cell activation”)

Defined by cognitive dysfunction/memory impairment, this endotype displayed elevated CD3^+^HLA-DR^+^% at month 3 (≈30% vs. ~20% in those without cognitive symptoms in cross-sectional comparisons), pointing to sustained T-cell activation despite clinical convalescence. This mirrors the cohort-level pattern of incomplete resolution of activation markers in a subset at one year.

Endotype C—Musculoskeletal (“Lymphopenic/immune-insufficiency”)

Comprised of participants reporting myalgia/arthralgia at 12 months and distinguished by lower total lymphocytes with reduced CD3^+^, CD3^+^CD4^+^, and CD19^+^ counts, suggesting a broader low-grade immune insufficiency rather than isolated activation.

Severity imprint and robustness

Qualitatively, the respiratory endotype was over-represented among survivors of critical illness, which also showed slower decline in activated T cells in our primary analyses, indicating that the magnitude of the initial immune insult may shape endotype expression during recovery.

Cluster memberships and distinguishing features were robust to sensitivity analyses using 6-month features alone and after covariate residualization. Cluster membership and key immune features stayed the same even after we adjusted all immune markers for age, sex, BMI, and initial disease severity, showing that these endotypes are not explained only by obesity-related chronic inflammation.

Nevertheless, these endotypes should be viewed as hypothesis-generating and warrant validation in larger, external cohorts.

**Figure 3:**
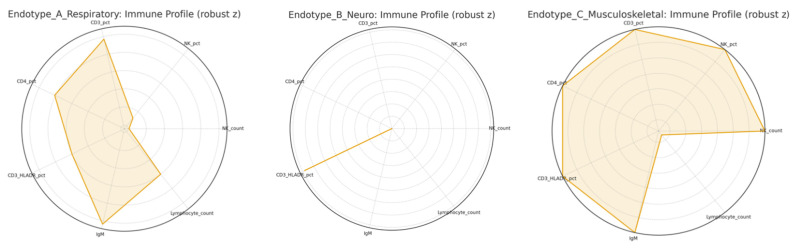
(exploratory). Radar plots of immune profiles per endotype. For each endotype (Respiratory, Neurocognitive, Musculoskeletal), spokes display the immune markers using the robust standardized differences; 0 indicates no difference vs. asymptomatic at the same timepoint.

**Figure 4:**
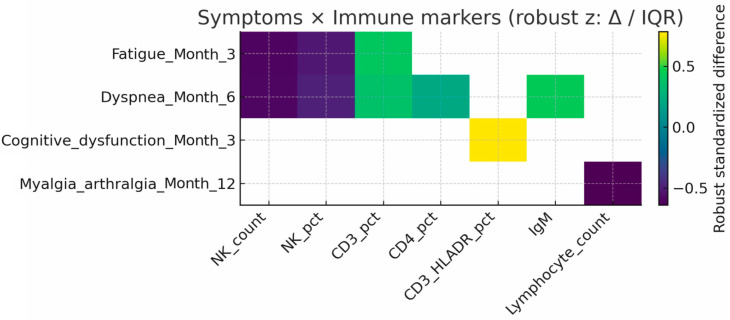
Immune markers associated with persistent symptoms at 3, 6, and 12 months. Group differences are shown for selected markers associated with fatigue, dyspnea, and musculoskeletal symptoms at follow-up.

Detailed analyses are shown in Figure 3 and Figure 4.

### 3.5. Influence of Disease Severity

When immune recovery was analyzed based on the severity of the initial illness, distinct patterns emerged. Patients who had been critically ill showed a slower decline in activated T cells (CD3^+^HLA-DR^+^) and more prolonged suppression of both CD3^+^CD4^+^ and CD3^+^CD8^+^ T cells compared to those with moderate or severe illness. Detailed changes in lymphocyte subpopulations stratified by disease severity are provided in Supplementary Materials S1–S3.

When immune parameters were stratified by initial disease severity (Table 4), patients recovering from critical COVID-19 maintained significantly higher IgM levels compared to those with severe disease across all timepoints: month 3 (1.01 vs. 0.75 g/L, *p* = 0.033), month 6 (0.96 vs. 0.60 g/L, *p* = 0.040), and month 12 (0.92 vs. 0.45 g/L, *p* = 0.044). IgG levels were also higher in the critical group at month 3 (11.67 vs. 10.27 g/L, *p* = 0.031).

Complement C3c increased across all severity groups but was more pronounced in severe and critical survivors.

## 4. Discussion

This 12-month prospective study demonstrates that clinical recovery after severe COVID-19 follows distinct immune trajectories. While most patients experienced substantial symptom improvement, immunological recovery showed heterogeneous patterns. T-cell activation normalized as expected, whereas complement C3c levels rose by ~10% and serum IgA by 32%. These findings reveal a dissociation between clinical and immune normalization, with implications for understanding long COVID pathophysiology. Our data suggest that persistent symptoms correspond to distinct immune endotypes: respiratory complaints linked to complement activation and NK cell deficits, while neurocognitive impairment associated with sustained T-cell activation. This heterogeneity implies that long COVID represents a spectrum of immune recovery pathways rather than a single syndrome.

Our findings align with previous studies reporting substantial but incomplete recovery within the first year after hospitalization [8,21,22,23]. At 12 months, nearly one quarter of patients still reported persistent symptoms, with dyspnea particularly common among those with critical illness. This severity gradient is consistent with studies linking initial disease severity to prolonged recovery [10,21]. Epidemiological studies show that even non-hospitalized individuals can develop persistent fatigue, cognitive impairment, and exercise intolerance, highlighting the broad spectrum of post-acute sequelae of SARS-CoV-2 infection (PASC) [24,25].

From an immunological perspective, severe COVID-19 is characterized by profound immune perturbations, including lymphopenia, T-cell hyperactivation, and disordered innate immunity. While markers of T-cell activation gradually decline over time, a subset of patients—particularly those recovering from critical illness—continue to exhibit activated T-cell populations months after infection [5,26,27,28,29]. Our observation of incomplete T-cell resolution in critically ill survivors aligns with these reports. Persistent dysregulation of adaptive immunity and lack of coordinated T- and B-cell recovery have been directly associated with ongoing long COVID symptoms. We observed declining activated CD3^+^HLA-DR^+^ T cells (20% to 13%), consistent with resolution of hyperactivation. However, approximately 20% of patients maintained elevated activated T-cell percentages at one year, particularly those with critical disease.

NK cells have emerged as another critical component in post-COVID trajectories. Reports consistently describe persistent functional impairment and reduced NK cell counts in severe and long COVID [30,31,32,33]. In our study, NK-cell counts remained stable at the group level, but individual-level analyses revealed persistent deficits in ~25% of patients. Patients with fatigue or dyspnea had lower NK-cell counts, consistent with reports linking NK-cell dysfunction to symptom persistence and supporting the hypothesis that impaired NK-cell surveillance contributes to long COVID pathogenesis [13,14,32,33]. These cross-sectional associations may be bidirectional: persistent inflammation may drive symptoms, but symptoms (e.g., reduced physical activity) may also exacerbate immune dysregulation.

Complement activation represents one of the most reproducible immune signatures of COVID-19 severity [12]. Activation at the C3 level has been implicated in acute respiratory failure and adverse outcomes [34,35]. Longitudinal analyses confirm that complement dysregulation can persist well into convalescence, with evidence of ongoing alternative pathway engagement and thromboinflammation in long COVID [36]. Our finding of progressively rising C3c levels despite clinical improvement aligns with this literature and suggests that complement remains pathologically active long after viral clearance. The progressive rise in C3c may drive persistent endothelial dysfunction and microthrombosis, as previously linked to complement dysregulation in COVID-19 [36], potentially explaining why a subset of patients continues to experience cardiopulmonary limitations despite clinical improvement. The therapeutic relevance of this pathway is underscored by promising early data on C3 and C5 inhibition in severe COVID-19.

Humoral responses also follow divergent patterns. While neutralizing antibodies and IgG responses undergo maturation and stabilization over time, mucosal immunity remains abnormally engaged. Several groups have reported sustained or rising IgA concentrations for up to a year post-infection [14,15,37]. Our study demonstrated a 32% increase in IgA levels over 12 months, suggesting prolonged mucosal immune activation. This may reflect persistent antigenic stimulation at mucosal surfaces, as recent studies have detected viral proteins in intestinal and respiratory mucosa months after infection, supporting the hypothesis of tissue reservoirs that sustain mucosal immune activation. Interestingly, other cohorts have noted diminished virus-specific IgA responses in patients with persistent symptoms, underscoring heterogeneity in mucosal immune recovery [38].

In an exploratory, unsupervised analysis integrating symptoms with immune features, we identified three reproducible endotypes: a respiratory profile linking dyspnea/exertional intolerance with NK-cell suppression, persistent T-cell activation, and complement involvement (early higher C3c, later heterogeneity); a neurocognitive profile marked by elevated CD3^+^HLA-DR^+^; and a musculoskeletal profile with lymphopenia spanning T- and B-cell compartments. These patterns align with our primary cross-sectional associations and support the concept that long-COVID represents a spectrum of immune recovery pathways, potentially requiring endotype-tailored monitoring and interventions (e.g., complement-targeted strategies or approaches to restore NK function). These associations are observational and hypothesis-generating; they do not establish causal relationships between immune dysregulation and long COVID. Prospective validation in larger cohorts with functional assays is needed.

Recent studies suggest that persistent viral antigens, particularly the SARS-CoV-2 spike protein, may remain detectable in circulation or immune cells for many months. This reservoir of viral material is hypothesized to sustain immune activation, providing a potential mechanistic link between antigen persistence and chronic inflammation in long COVID [30,32,33].

The more profound immune alterations in critically ill patients suggest that the magnitude of the initial immune insult determines both short-term outcomes and the trajectory of immune recovery. This ‘immune scarring’ may help stratify patients at highest risk of developing long COVID. Survivors of critical illness showed slower decline of activated T cells (CD3^+^HLA-DR^+^), more prolonged suppression of CD4/CD8 subsets, persistently higher IgM across 3, 6, and 12 months, and a more pronounced rise in C3c compared with less severe cases. These patterns indicate durable remodeling of adaptive and humoral responses after overwhelming activation—conceptually analogous to post-critical-illness immune changes described after ARDS or sepsis. Initial severity appears to determine the slope and set-point of recovery (endotype expression, complement tone, and NK/T-cell balance), not just its timing, underscoring the need for severity-stratified follow-up and immunomonitoring.

There are parallels to post-viral fatigue syndromes. The immune pattern we observe—NK-cell dysfunction, persistent T-cell activation, and complement dysregulation—echoes post-infectious fatigue states. Meta-analyses in myalgic encephalomyelitis/chronic fatigue syndrome (ME/CFS) show markedly reduced NK cytotoxicity versus healthy controls, a robust, reproducible abnormality [39,40,41]. Complement involvement is also reported in fatigue syndromes (e.g., exercise-induced C4a rises in CFS) and is a recognized amplifier of respiratory viral pathology such as influenza [42]. Long-term chronic fatigue has been documented in SARS-CoV-1 survivors years after illness, supporting a shared, severity-imprinted immune footprint across viruses [43].

Both our data and the broader literature indicate that clinical recovery and immune restoration do not always proceed in parallel. Long-term alterations in T-cell activity, NK cell competence, mucosal IgA responses, and complement regulation provide a biological rationale for symptom persistence in a subset of survivors. Recognition of these patterns highlights the need for extended follow-up, immune monitoring, and consideration of targeted therapeutic strategies aimed at correcting sustained immune dysregulation.

## 5. Study Limitations

This study has several limitations that should be considered when interpreting results. The absence of healthy controls limits our ability to distinguish COVID-specific changes from general post-critical illness recovery patterns. Reinfections during follow-up were not systematically captured. Symptom assessment relied on structured interviews rather than validated functional measures, which may have introduced subjective bias and limited sensitivity in detecting subtle impairments. Additionally, some subgroup analyses had limited statistical power due to small sample sizes, particularly when stratifying by disease severity, which restricted the robustness of associations between immune markers and persistent symptoms. Laboratory data completeness varied across timepoints due to missed visits and technical issues, potentially introducing bias. Moreover, all patients were unvaccinated, meaning the findings may not be generalizable to post-vaccination or breakthrough infection cohorts. Also, given that >90% of our cohort was overweight or obese and that no BMI-matched non-COVID control group was included, we cannot definitively separate obesity-related chronic inflammatory changes from COVID-19–related immune dysregulation. Our findings should therefore be interpreted primarily as longitudinal trajectories within survivors of severe COVID-19 rather than as specific to SARS-CoV-2 when compared with obese individuals without prior infection.

Because nearly all participants were overweight or obese and the number of normal-weight individuals was small, we were underpowered to perform robust stratified analyses by BMI category; thus, residual confounding by obesity-related metabolic inflammation cannot be excluded.

Finally, mechanistic insights into the biological drivers of persistent immune activation remain limited, as functional assays (e.g., NK cytotoxicity, T-cell exhaustion markers, complement activation fragments) were not included. Despite these limitations, our longitudinal design with comprehensive immune profiling provides valuable insights into post-COVID recovery patterns.

## 6. Clinical and Research Implications

This study helps to clarify immune recovery after severe COVID-19, showing that clinical improvement often outpaces immune normalization; therefore, symptom resolution is not equivalent to full physiological recovery. Next research steps include evaluating whether immune markers can predict clinical outcomes and guide individualized follow-up strategies, and investigating underlying mechanisms—particularly sustained C3 activation and rising IgA—in larger, multicenter cohorts including healthy controls. Although this study did not include a non-COVID pneumonia or healthy control group, which limits differentiation between SARS-CoV-2–specific immune alterations and general post-critical illness recovery patterns, comparative studies with influenza and bacterial pneumonia cohorts are currently underway within our institution. These investigations will help determine whether the complement and IgA trajectories observed here are unique to COVID-19.

## 7. Conclusions

One year after hospitalization for moderate-to-critical COVID-19, most patients achieved substantial clinical improvement, yet a quarter continued experiencing fatigue or dyspnea. Immunological recovery was incomplete and heterogeneous: activated T cells declined as expected, but C3c and IgA rose progressively, and approximately 25% of patients showed persistent NK-cell deficits. This dissociation between clinical and immunological recovery indicates that symptom resolution does not equate to immune restoration.

These immune alterations—particularly sustained complement activation, elevated mucosal IgA, and NK-cell dysfunction—may contribute to persistent symptoms and warrant further mechanistic investigation. Our findings support the implementation of severity-stratified, extended follow-up protocols incorporating immune monitoring. Future research should validate these immunological markers as potential tools for risk stratification and guide the development of targeted interventions for post-COVID populations.

## Figures and Tables

**Table 1:** Prevalence of post-COVID-19 symptoms at 3, 6, and 12 months post-discharge, N (%).

Post-COVID-19 Symptom	Month 3	Month 6	Month 12	3 vs. 6 Months	3 vs. 12 Months	6 vs. 12 Months
Fatigue	66 (70.97)	37 (39.78)	23 (24.73)	<0.001	<0.001	0.001
Dyspnoea	76 (81.72)	49 (52.69)	24 (25.81)	<0.001	<0.001	<0.001
Reduced exercise tolerance	66 (70.97)	47 (50.54)	23 (24.73)	0.001	<0.001	<0.001
Myalgia/arthralgia	19 (20.43)	12 (12.90)	14 (15.05)	0.092	0.424	0.824
Cardiac symptoms	11 (11.83)	2 (2.15)	3 (3.23)	0.004	0.057	1.000
Insomnia/anxiety	11 (11.83)	9 (9.68)	9 (9.68)	0.791	0.815	1.000
Cognitive dysfunction/memory impairment	9 (9.68)	10 (10.75)	7 (7.53)	1.000	0.754	0.453
Miscellaneous	33 (35.48)	21 (22.58)	15 (16.13)	0.043	0.002	0.180

McNemar’s test was used to assess within-subject changes in symptom presence between two time points.

**Table 2:** Absolute number and percentage of lymphocyte populations of patients at 3, 6 and 12 months post-discharge.

Parameter	N	Absolute Number (Median, IQR), Cells/mm^3^	Percentage (Median, IQR)
Month 3			
Lymphocytes	93	2079(1636–2788)	36 (30–45) a
CD3^+^	88	1462(1145–2171.50) a	72 (68–77.75) a,b
CD3^+^CD4^+^	93	818 (643–1082.50)	39 (33–47)
CD3^+^CD8^+^	93	632 (389.5–984.50) b	31 (21–39.5) a,b
CD4/CD8	92	1.2 (0.85–2.20)	
NK	93	325 (194.50–506.50)	16 (10–21.5)
CD19^+^	93	184 (135–255.50)	8 (6–11)
CD3^+^HLA-DR^+^	88	413 (242.75–689.25) a,b	20 (14–26.75) a,b
Month 6			
Lymphocytes	93	2014(1682.50–2518)	34 (27–42) a
CD3^+^	92	1438(1090.75–1884.50) a	71 (65–75.75) a
CD3^+^CD4^+^	92	791.50(598.25–1029.25)	39.50 (34–43.50)
CD3^+^CD8^+^	93	619 (430–928.50) c	31 (21–40) a,c
CD4/CD8	93	1.30 (0.95–2.30)	
NK	93	320 (184–499)	17 (11–23.25)
CD19^+^	93	172 (124.50–247.50)	9.67 (6.70–12)
CD3^+^HLA-DR^+^	92	337.50 (192–530) a,c	16 (12–22) a,c
Month 12			
Lymphocytes	85	2016(1565–2 530.5)	34.80 (29–42)
CD3^+^	84	1415.5(1025.75–1926)	72 (65–78) b
CD3^+^CD4^+^	85	778 (623–1054.50)	40 (35.5–46)
CD3^+^CD8^+^	85	572 (405–891) b,c	29 (22–39) b,c
CD4/CD8	85	1.26 (0.92–2.25)	
NK	85	321 (174.50–543)	15 (10.65–23.45)
CD19^+^	85	177 (131.50–248.50)	9 (6.25–12)
CD3^+^HLA-DR^+^	82	285 (162.25–456) b,c	13 (9.98–20) b,c

a—*p* < 0.05 for comparison between month 3 vs. month 6. b—*p* < 0.05 for comparison between month 3 vs. month 12. c—*p* < 0.05 for comparison between month 6 vs. month 12. Wilcoxon signed-rank test was used for all time-point comparisons.

**Table 3:** Complement and immunoglobulins (C3c, C4, IgA, IgG, IgM, IgE) at 3, 6, and 12 months post-discharge.

Parameter	Month 3	Month 6	Month 12
C4, g/L	0.25 (0.22–0.29) a	0.26 (0.19–0.29) a	0.24 (0.20–0.30)
C3c, g/L	1.23 (1.10–1.39) a,b	1.30 (1.05–1.39) a,c	1.35 (1.17–1.51) b,c
IgA, g/L	2.06 (1.53–2.67) a,b	2.42 (1.81–3.74) a	2.72 (1.82–3.35) b
IgG, g/L	11.14 (9.54–12.39)	11.16 (9.98–12.94)	11.72 (10.11–12.49)
IgM, g/L	0.92 (0.61–1.35)	0.77 (0.47–1.34)	0.69 (0.44–1.37)
IgE, g/L	20.50 (12.50–52.80)	21.90 (13.80–112.60)	22.10 (10.05–167.90)

a—*p* < 0.05 for comparison between month 3 vs. month 6. b—*p* < 0.05 for comparison between month 3 vs. month 12. c—*p* < 0.05 for comparison between month 6 vs. month 12. Wilcoxon signed-rank test was used for all time-point comparisons.

**Table 4:** Complement and immunoglobulins by initial severity (moderate, severe, critical) at 3, 6, and 12 months.

Parameter	Moderate COVID-19	Severe COVID-19	Critically Severe COVID-19
Month 3			
C4, g/L	0.28 (0.22–0.29)	0.25 (0.20–0.28)	0.25 (0.22–0.32)
C3c, g/L	1.26 (1.13–1.46)	1.29 (1.12–1.40)	1.20 (1.08–1.39)
IgA, g/L	1.61 (1.29–2.33)	2.37 (1.45–3.02)	2.07 (1.68–2.52)
IgG, g/L	10.67 (9.51–12.27)	10.27 (9.27–11.49) *	11.67 (10.07–12.85) *
IgM, g/L	0.96 (0.67–1.35)	0.75 (0.44–1.24) *	1.01 (0.73–1.46) *
IgE, g/L	14.40 (8.08–28.25)	21.00 (12.50–86.60)	32.50 (13.95–51.58)
Month 6			
C4, g/L	0.24 (0.20–0.31)	0.27 (0.20–0.30)	0.26 (0.18–0.29)
C3c, g/L	1.24 (0.86–1.38)	1.34 (1.26–1.52)	1.25 (1.04–1.38)
IgA, g/L	2.33 (1.54–4.22)	2.37 (1.42–3.54)	2.57 (2.18–3.71)
IgG, g/L	11.26 (10.30–12.55)	10.71 (9.70–13.40)	11.50 (9.95–13.06)
IgM, g/L	0.93 (0.46–1.59)	0.60 (0.30–0.78) *	0.96 (0.52–1.77) *
IgE, g/L	18.70 (8.40–193.73)	48.85 (17.08–213.23)	21.90 (10.60–86.85)
Month 12			
C4, g/L	0.26 (0.24–0.31)	0.25 (0.17–0.31)	0.24 (0.19–0.33)
C3c, g/L	1.38 (1.18–1.47)	1.47 (1.06–1.59)	1.30 (1.17–1.53)
IgA, g/L	2.21 (1.77–3.65)	3.20 (1.82–4.37)	2.58 (1.80–3.23)
IgG, g/L	11.46 (10.81–12.31)	11.86 (10.05–12.24)	11.67 (9.15–13.86)
IgM, g/L	0.54 (0.37–1.19)	0.45 (0.40–1.13) *	0.92 (0.66–2.62) *
IgE, g/L	33.65 (18.15–186.60)	52.10 (14.18–431.90)	14.80 (6.60–58.50)

*—*p* < 0.05 for comparison between severe and critically severe COVID-19 groups. Mann–Whitney U test was used for group comparisons.

## Data Availability

The original contributions presented in the study are included in the article; further inquiries can be directed to the corresponding authors.

## Supplementary Materials

The following supporting information can be downloaded at: https://www.mdpi.com/article/10.3390/v17121610/s1, Table S1. Absolute number and percentage of lymphocyte populations of patients recovered from moderate COVID-19 at 3, 6 and 12 months post-discharge. Table S2. Absolute number and percentage of lymphocyte populations of patients recovered from severe COVID-19 at 3, 6, and 12 months post-discharge. Table S3. Absolute number and percentage of lymphocyte populations of patients recovered from critically severe COVID-19 at 3, 6 and 12 months post-discharge.
