# Supplementary material for: Clinical and Immunological Recovery Trajectories in Severe COVID-19 Survivors: A 12-Month Prospective Follow-Up Study
Source: Viruses. 2025 Dec 12;17(12):1610. doi: 10.3390/v17121610 (PMC12737718; doi:10.3390/v17121610)
Supplement: Supplementary file 1 [file viruses-17-01610-s001.zip › viruses-3932282-supplementary.pdf]

viruses

MDPI

Table S1. Absolute number and percentage of lymphocyte populations of patients recovered from moderate COVID-19 at 3,6 and 12 months post-discharge.

Parameter	N	Absolute Number (Median, IQR), cells/mm^3	Percentage (Median, IQR)
		Month 3	
Lymphocytes	17	1 938 (1 578.50 - 2 305)	35 (30.50 - 41)
CD3$^+$	16	1 304.50 (1 149.75 - 1 604.50)	70 (66.50 - 75)
CD3$^+$CD4$^+$	17	765 (674 - 965)	48 (32.50 - 53) a
CD3$^+$CD8$^+$	17	515 (313 - 713.50)	23 (17 - 35)
CD4/CD8	17	2.30 (0.85 - 3.70) a	
NK	17	291 (142.50 - 351.50)	14 (8.50 - 18) a,b
CD19$^+$	17	230 (138.50 - 286)	12 (6.50 - 14.50)
CD3$^+$HLA-DR+	16	190.50 (126 - 447.50)	10 (7.50 - 19.50)
		Month 6	
Lymphocytes	17	1 692 (1 500 - 2 204)	32 (28 - 37.50)
CD3$^+$	17	1 240 (918.50 - 1 552)	72 (65 - 72.65)
CD3$^+$CD4$^+$	16	724.50 (465.75 - 1 084)	41 (31.50 - 51.50) a
CD3$^+$CD8$^+$	17	468 (308 - 645)	23 (19 - 38.50) c
CD4/CD8	17	1.40 (0.86 - 2.55) a	
NK	17	264 (185 - 413)	13.40 (12 - 19.50) a
CD19$^+$	17	164 (138.50 - 286.50)	11 (8.35 - 13.70)
CD3$^+$HLA-DR+	17	193 (147 - 352.50)	13 (8.85 - 20) c
		Month 12	
Lymphocytes	15	1 845 (1 540 - 2 485)	32 (28 - 39)
CD3$^+$	14	1 296 (1 071.50 - 1 785.25)	70.50 (63.50 - 75.25)
CD3$^+$CD4$^+$	15	875 (444 - 1 093)	45 (34 - 51)
CD3$^+$CD8$^+$	15	513 (332 - 777)	24 (18 - 37) c
CD4/CD8	15	2.10 (0.87 - 3.10)	
NK	15	294 (185 - 348)	14 (10.30 - 21) b
CD19$^+$	15	211 (137 - 341)	11 (8.80 - 15)
CD3$^+$HLA-DR+	14	210.50 (125 - 351.25)	9.45 (6.83 - 16.25) c

a – $p<0.05$ for comparison between month 3 vs month 6

b – $p<0.05$ for comparison between month 3 vs month 12

c – $p<0.05$ for comparison between month 6 vs month 12

Wilcoxon signed-rank test was used for all time-point comparisons.

Table S2. Absolute number and percentage of lymphocyte populations of patients recovered from severe COVID-19 at 3, 6, and 12 months post-discharge.

Parameter	N	Absolute Number (Median, IQR), cells/mm^3	Percentage (Median, IQR)
		Month 3	
Lymphocytes	32	2 142 (1 551.75 - 2 921.75)	36.50 (28.25 - 45)
$CD3^+$	30	1 602 (1 009 - 2 223.50)	71 (67.25 - 78.25) a
$CD3^+CD4^+$	32	829 (642 - 1 204)	40 (33 - 48.50)
$CD3^+CD8^+$	32	688.50 (309 - 1 187)	31 (23 - 39.25)
CD4/CD8	32	1.25 (0.92 - 2.18)	
NK	32	341.50 (188.50 - 495.25)	16 (10.25 - 24.25)
$CD19^+$	32	182.50 (127.50 - 253.25)	8 (6 - 10.75)
$CD3^+$HLA-DR+	30	449 (201.75 - 686)	20.50 (11 - 28.50) b
		Month 6	
Lymphocytes	32	2 106.50 (1 618.50 - 2 898)	34 (26.25 - 42.75)
$CD3^+$	32	1 566.50 (977.25 - 2 049.75)	70 (60.50 - 77) a
$CD3^+CD4^+$	32	836 (588.25 - 1 074)	39.50 (34.25 - 42.75)
$CD3^+CD8^+$	32	692 (438.25 - 968.25)	31 (19.50 - 40)
CD4/CD8	32	1.23 (0.94 - 2.30)	
NK	32	418 (198.75 - 618.50)	19 (9.75 - 27)
$CD19^+$	32	171.50 (112.50 - 248)	8.50 (6.10 - 10.75)
$CD3^+$HLA-DR+	32	393 (197.25 - 586.25)	16 (12.48 - 25) c
		Month 12	
Lymphocytes	30	2 272 (1 625.50 - 2 958.75)	35.30 (31.50 - 45.25)
$CD3^+$	30	1 488.50 (984 - 2 236)	71 (59.50 - 79.25)
$CD3^+CD4^+$	30	795 (622.50 - 1 076.25)	38 (32.75 - 45.25)
$CD3^+CD8^+$	30	622 (369.75 - 1 103.50)	29 (21.50 - 41.25)
CD4/CD8	30	1.20 (0.88 - 2.35)	
NK	30	400.50 (182.50 - 663.25)	17.50 (10.60 - 27.25)
$CD19^+$	30	175 (120 - 269.50)	8 (6.38 - 11.25)
$CD3^+$HLA-DR+	29	338 (187 - 665.50)	14 (10 - 21.50) b,c

a – $p<0.05$ for comparison between month 3 vs month 6

b – $p<0.05$ for comparison between month 3 vs month 12

c – $p<0.05$ for comparison between month 6 vs month 12

Wilcoxon signed-rank test was used for all time-point comparisons.

viruses MDPI

Table S3. Absolute number and percentage of lymphocyte populations of patients recovered from critically severe COVID-19 at 3,6 and 12 months post-discharge.

Parameter	N	Absolute Number (Median, IQR), cells/mm^3	Percentage (Median, IQR)
		Month 3	
Lymphocytes	44	2 320 (1 723.50 - 2 934.25)	37 (30.25 - 45.50)
CD3$^+$	42	1 607.50 (1 177.25 - 2 251.25) a,b	72 (67.75 - 78) a,b
CD3$^+$CD4$^+$	44	812 (617 - 1 088.75)	38 (32.25 - 43) a,b
CD3$^+$CD8$^+$	44	697.50 (427.25 - 1 157.75) a,b	33.50 (24.25 - 40.75) a,b
CD4/CD8	43	1.10 (0.80 - 1.70) a,b	
NK	44	348 (205.25 - 560.50) a,b	16.50 (10.48 - 23)
CD19$^+$	44	181.50 (134 - 232.75)	8 (6.25 - 10.75) b
CD3$^+$HLA-DR+	42	491.50 (301 - 796.50) a,b	22 (18.75 - 26) a,b
		Month 6	
Lymphocytes	44	1 980.50 (1 751.25 - 2 765.75)	36.50 (27.50 - 42.75)
CD3$^+$	43	1 428 (1 168 - 1 975) a	72 (66 - 78) a
CD3$^+$CD4$^+$	44	809.50 (644 - 983.25)	38.50 (34.25 - 43) a
CD3$^+$CD8$^+$	44	589.50 (451.50 - 955.50) a	31 (25.25 - 40) a
CD4/CD8	44	1.26 (0.99 - 1.70) a	
NK	44	311 (162.25 - 495.25) a	15.50 (9.40 - 22.50)
CD19$^+$	44	196.50 (118 - 240.75)	9.50 (6.25 - 11.75)
CD3$^+$HLA-DR+	43	344 (212 - 530) a	17 (12 - 22) a,c
		Month 12	
Lymphocytes	40	2 058 (1 506.75 - 2 458.25)	35 (26 - 42)
CD3$^+$	40	1 385 (1 061.25 - 1 835) b	73 (65 - 78) b
CD3$^+$CD4$^+$	40	711.50 (627.50 - 954.75)	39.50 (36 - 45) b
CD3$^+$CD8$^+$	40	604.50 (458.75 - 861.75) b	29.50 (25.25 - 39) b
CD4/CD8	40	1.23 (0.94 - 1.64) b	
NK	40	306.50 (160.25 - 444) b	14 (10.25 - 21.55)
CD19$^+$	40	176.50 (128 - 241.25)	8.50 (6 - 12) b
CD3$^+$HLA-DR+	39	282 (172 - 450) b	14 (12 - 19) b,c

a – $p<0.05$ for comparison between month 3 vs month 6

b – $p<0.05$ for comparison between month 3 vs month 12

c – $p<0.05$ for comparison between month 6 vs month 12

Wilcoxon signed-rank test was used for all time-point comparisons.
